# Putting the prime in priming: Using prime processing behavior to predict target structural processing

**DOI:** 10.3758/s13423-025-02643-3

**Published:** 2025-01-30

**Authors:** Kristen M. Tooley, Laurel Brehm

**Affiliations:** 1 Department of Psychology, Texas State University, 601 University Drive, San Marcos, TX 78666 USA; 2 University of California, Santa Barbara, CA USA

**Keywords:** Structural priming, Processing relationships, Language comprehension

## Abstract

Structural priming effects are widespread and heavily relied upon to assess structural representation and processing. Whether these effects are caused by error-driven implicit learning, residual activation, a combination of these, or some other learning mechanism remains to be established. The current study used preexisting data and a novel data analysis approach that links processing at the prime to later processing at the target to better understand the nature of structural priming. This novel analytic approach was applied to total reading times from a previously published structural priming study in comprehension, which provided processing measures of the structurally critical regions of prime reduced-relative clause sentences. These were then used as predictors in a series of hierarchical linear models where analogous processing measures at the target sentence regions served as outcome variables. Separate sets of models were run for prime–target pairs that had the same structure (i.e., abstract priming) and those that had the same structure and initial verb (i.e., a lexical boost). Prime-to-target processing relationships were observed for both types of prime–target pairs, but showed very different patterns. This provides support for the claim that abstract priming effects and the lexical boost are caused by different mechanisms. Additionally, the observed effects were positive and so do not support the error-driven learning prediction that processing difficulty at the prime should lead to greater facilitation at the target. Overall, this novel method provides a new tool for investigating structural priming and processing.

**Supplementary Information:**

The online version contains supplementary material available at 10.3758/s13423-025-02643-3.

Critical to any model of language processing is an explanation of how structural knowledge is represented and used. Foundational work by Bock and colleagues (e.g., Bock, 1986; Bock & Loebell, 1990) systematically showed *structural priming* in language production. Participants were primed to describe a novel picture with the syntactic structure of a recently heard/repeated sentence. Such effects have been replicated and extended within and across modalities (e.g., Segaert et el., 2013; Tooley & Bock, 2014), within and across languages in bilinguals (see Van Gompel & Arai, 2018), in children (see Messenger, 2021), in amnesiacs (Ferreira et al., 2008), in corpus studies (e.g., Gries, 2005), and in cognitive models (e.g., Chang et al., 2006; Reitter et al., 2011). The pervasiveness of structural priming provides strong evidence for fully independent, abstract structural representations (see Branigan & Pickering, 2017; Pickering & Ferreira, 2008, for reviews).

Most structural priming studies use production paradigms similar to J. K. Bock (1986), which are consistently sensitive to priming effects under varying contexts. Structural priming is also observed during sentence comprehension, where effects are typically measured using reading or looking times. For example, reading times on “The girl tossed the blanket on the bed into the laundry this morning” suggest readers initially assume “on the bed” is the location the blanket was tossed. This garden-path effect is reduced (shorter reading times) after reading a prime sentence with the same structure (Traxler, 2008). Similarly, eye movements to an array of objects on hearing “send the” reveal the expected structural interpretation as either a double object (e.g., to the frog for “send the frog the gift”) or a prepositional object (e.g., to the gift for “send the gift to the frog”), with expectations affected by the preceding prime sentence’s structure (Thothathiri & Snedeker, 2008). Both of these studies observed abstract structural priming (no shared content words between primes and targets) in sentence comprehension.

While abstract structural priming is sometimes more difficult to detect in comprehension than production, this is likely due to differences in structures and paradigms used across modalities (see Tooley, 2023, for a review). When methodological differences are controlled, structural priming effects in comprehension and production are not meaningfully different (e.g., Pickering et al., 2013; Segaert et al., 2013; Tooley & Bock, 2014). Thus, the same underlying mechanisms produce these effects in both modalities, and production and comprehension paradigms are valid for studying structural priming. This is critical given debates surrounding the underlying mechanisms of structural priming.

Activation accounts suggest structural priming reflects residual activation for recently used structural representations and the words (particularly verbs) participating in them (Malhotra et al., 2008; Pickering & Branigan, 1998). These accounts are similar to traditional lexical priming accounts (e.g., Meyer & Schvaneveldt, 1971) but are applied to structural representations attached to lemma-level representations (e.g., Roelofs, 1993). When a prime structure is encountered, activation of structural representations and linked lemma representations persists for a short time, facilitating reuse/re-access of the primed structure. When the verb from the prime is re-encountered in the target, residual activation for the structure combines with residual activation for the link between verb and structure, leading to a boosted tendency to reuse/re-access the primed structure. This “lexical boost” is a widely observed feature of structural priming (see Mahowald et al., 2016). Activation-based models, therefore, parsimoniously account for abstract structural priming effects (priming from structure alone) and the lexical boost (priming from structure and lexical overlap).

Yet findings that structural priming accumulates over multiple exposures and can be detected a week after mass exposure (Kaschak et al., 2011, 2014) make an activation-based mechanism unlikely. An alternate mechanistic account based on implicit learning or adaptation (e.g., Chang et al., 2006, 2012) suggests that planning/parsing a structure acts like procedural learning within the language processing system, making the encountered structure easier to construct and more prepotent. Computational models that modulate structural weightings based on an error signal from the degree of match/mismatch of the expected and experienced structures have successfully simulated structural priming effects (Chang et al., 2006; Jaeger & Snider, 2013). These models explain abstract structural priming, accumulation of priming/learning, and the inverse frequency effect: greater structural priming for less frequent structures due to larger error signals during prime exposure (Fine & Jaeger, 2013).

Unlike activation-based accounts, however, implicit learning models do not predict a lexical boost. To address this shortcoming, some learning accounts suggest the lexical boost is caused by explicit memory for the wording of the prime (see Chang et al., 2012), not the implicit learning driving abstract priming. Other learning accounts suggest the lexical boost stems from activation changes within the short-term memory system (see Reitter et al., 2011, for an associative learning account). Neither mechanistic account can straightforwardly explain the entire body of structural priming effects. Making progress in this mechanistic debate therefore requires a novel approach.

One unexplored approach involves utilizing both prime and target processing to better understand how initial exposure leads to later structural facilitation. This approach is possible for comprehension studies with ongoing processing measures (e.g., eye-tracking, electroencephalogram, or functional magnetic resonance imaging), and relies on the fact that experience with the prime is what *causes* later facilitation on the target. This is essential for structural priming and true regardless of mechanistic account. Under the residual activation account, encountering the *prime* structure (with a specific verb) causes structural elements to be activated, which then facilitates target processing. Under the implicit learning account, processing of the *prime* structure leads to weighting changes within the language processing system that yield structural processing changes at the target. Thus, investigating processing during prime exposure and its *relationship* to subsequent target processing provides critical evidence for the cause of structural priming. This idea is reminiscent of theoretical advances in memory research that arose from looking back at encoding for items that were later remembered versus those that were not (e.g., Brewer et al., 1998). This approach could similarly shed new light on the nature of structural priming effects and the mechanisms that support them.

Using processing measures at different sentence regions is also important for establishing *how* the prime exposure leads to later target facilitation. Sentence processing unfolds incrementally (Allopenna et al., 1998; Eberhard et al., 1995), so prime–target processing relationships may exist at all sentence regions where there is structural overlap. Alternatively, it may be that processing specific regions of the prime, such as structurally critical regions, is more strongly associated with processing the target structure. Critically, the predicted pattern and direction of prime–target processing relationships differ across mechanistic accounts.

An error-driven implicit learning account suggests that the error signal generated during prime processing leads to weighting changes that produce priming on the target. This account predicts an inverse relationship between prime and target processing where more difficulty in prime processing leads to less difficulty processing the target. However, positive correlations between prime and target processing are predicted by the residual activation account (e.g., Pickering & Branigan, 1998) and associative learning accounts (e.g., Reitter et al., 2011). In a comprehension framework, these accounts suggest that successfully parsing the prime structure leads to strong activation for that structure’s representation, which then persists during target processing. Primes where participants initially favored a different structural interpretation would have longer total reading times and less relative activation of the primed structure representation, resulting in less facilitation (and longer reading times) of the targets. This is caused by short-lived (residual activation) or long-lived (associative learning) increases in structural activation.

The different mechanistic accounts also imply differences in the processing relationships when only the structure is shared (abstract priming) versus when both structure and verb are shared between prime and target (lexical boost). The residual activation account proposes that both abstract priming and the lexical boost come from the same residual activation mechanism. This account (e.g., Pickering & Branigan, 1998) therefore predicts prime–target processing relationships will be *qualitatively* very similar, emerging at the same structural regions, regardless of whether prime and target share the same structure or the same structure and verb, but that the magnitude of effects will vary. The implicit learning account suggests abstract effects are supported by an implicit learning mechanism but the lexical boost is driven by a separate explicit memory mechanism. This predicts a qualitatively different pattern when structure and verb overlap in primes than when just structure overlaps.

The current study explores prime–target processing relationships in a previously published structural priming study that observed abstract and lexically boosted priming effects during comprehension of adjacent sentences. Without this necessary precondition, we would have little assurance that observed processing relationships reflect structural priming mechanisms. For this reason, we have been selective in our choice of data set (Tooley, 2020) and use secondary data analysis specifically to 1) determine if there is a relationship between prime and target processing in conditions where structural priming effects have been observed, 2) describe the nature of this relationship (i.e., is it a negative relationship as one might expect from a strong error-driven learning mechanism?), and 3) determine where and when these prime–target processing relationships exist to further adjudicate the residual activation and implicit learning mechanistic accounts.

## Method

### Data

The included data consist of total fixation time measures in the verb, by-phrase, and spillover regions of prime and target reduced-relative clause (RRC) items in Experiment 2 of Tooley (2020). This dataset is publicly available at https://doi.org/10.18738/T8/A5L2EF. These data represent observations from 58 participants who silently read 48 yoked prime–target pairs and unrelated fillers. Target sentences were always RRC sentences (e.g., “*The boy pushed by the girl made a sexist remark*”). Prime sentences took one of four forms: 1) a main clause structure with a different initial verb from the target, 2) a locative structure with a by-phrase, and with the same verb from the target, (3) an RRC structure with a different verb from the target, or (4) an RRC structure with the same verb as the target (see sentences 1–4, below).

1. Main clause, different verb: *The child noticed the man who was going to the front of the line.*
2. Locative, same verb: *The child pushed by the man to get to the front of the line.*
3. RRC, different verb: *The child noticed by the man was going to the front of the line.*
4. RRC, same verb: *The child pushed by the man was going to the front of the line.*

Target: *The boy pushed by the girl made a sexist remark*

These sentences manipulate the structure and verb match between prime and target: Compared to the target, Prime 1 has a different structure and verb, Prime 2 has a different structure but same verb, Prime 3 has the same structure but different verb, and Prime 4 has the same structure and verb. This design allows us to investigate processing relationships between the primes and targets with varied structural and lexical overlap. Furthermore, both abstract priming and a lexical boost effect were observed in this data set, making it uniquely appropriate for investigating prime–target processing relationships during structural priming.

### Data processing

In eye-tracking research, total time represents a sum of all the time a sentence region was fixated during reading. This includes initial fixations and fixations after regressions from a later part of the sentence (i.e., re-reading). In order to equate reading times across items, the calculated total time data was first mean-centered for each individual item, separately for primes and targets, while maintaining by-participant trial-level data. This means that for each of the 48 primes and 48 targets, each participant’s raw total fixation times were transformed into mean-centered total fixation times (based on the mean for that item). This process was repeated for each of the three sentence regions of interest in the RRC—the verb, by-phrase, and spillover regions. The verb region contained the initial (past tense) verb in the sentence (e.g., *pushed* in the example target sentence, above). The by-phrase region included the word *by* and its following noun phrase (e.g., *by the girl* in the example sentence). Lastly, the spillover region included the two words following the by-phrase region (e.g., *made a* in the example sentence).

### Predictions

First, processing for a given structure should predict processing for a subsequent experience with that structure. This assesses the basic concept of structural priming: Prime processing is causally related to future (target) processing for a given structure. Secondly, if abstract priming effects are created by an error-driven learning mechanism, we predict a negative relationship between processing times at the prime and those at the target, since difficulty with prime processing should produce a larger error signal and thus increased learning. A residual activation mechanism does not have this feature and therefore would not predict a negative processing relationship between primes and targets. Finally, if the mechanisms driving abstract priming effects and the lexical boost are different (as proposed by the implicit learning account), we predict to find a different pattern of processing relationships between primes and targets when only the structure overlapped versus when the structure and verb overlapped between them. Conversely, if the same (residual activation) mechanism that produces abstract priming also produces the lexical boost, then we expect to see the same prime–target relationships emerge in the RRC-Different verb and RRC-Same verb conditions, but the magnitude of the effects should be greater for the RRC-Same verb primes.

### Priming analyses

The analyses included data from the RRC target sentence regions (outcome measures) and the RRC prime sentences (predictors). The analyses are grouped into three sets: those that model abstract priming effects by using different-verb RRC prime sentences as predictors (Models 1–3), those that model lexically boosted effects by using same-verb RRC prime sentences as predictors (Models 4–6), and those that model the other conditions from this experiment with a different structure as the target but with overlapping verb and by-phrase information (locatives) or not (main clauses) (see Models 7–11). The models in each analysis set use the three different *target* sentence regions as outcome measures to explore which aspects of prime processing have the greatest influence on later target processing. In all analyses, all prime sentence regions were used as fixed-effect predictors (see details below). Participant was included as a random intercept. Since the data was mean-centered by item, no by-item random effects were included in the models as their contribution would be null. All analyses were run in R (R Core Team, 2021), RStudio (Rstudio Team, 2020), and the *lme4* package (Version 1.1–31).

#### Abstract priming effects models:

1. RRC-Different Verb **Target Verb Time** ~ Intercept + β_1_PrimeVerbTime + β_2_PrimeBy-phraseTime + β_3_PrimeSpilloverTime + error
2. RRC-Different Verb **Target By-phrase Time** ~ Intercept + β_1_PrimeVerbTime + β_2_PrimeBy-phraseTime + β_3_PrimeSpilloverTime + error
3. RRC-Different Verb **Target Spillover Time** ~ Intercept + β_1_PrimeVerbTime + β_2_PrimeBy-phraseTime + β_3_PrimeSpilloverTime + error

#### Lexical boost effects models:

4. RRC-Same Verb **Target Verb Time** ~ Intercept + β_1_PrimeVerbTime + β_2_PrimeBy-phraseTime + β_3_PrimeSpilloverTime + error
5. RRC-Same Verb **Target By-phrase Time** ~ Intercept + β_1_PrimeVerbTime + β_2_PrimeBy-phraseTime + β_3_PrimeSpilloverTime + error
6. RRC-Same Verb **Target Spillover Time** ~ Intercept + β_1_PrimeVerbTime + β_2_PrimeBy-phraseTime + β_3_PrimeSpilloverTime + error

### Control analyses

In order to ensure that observed relationships truly reflect processing associated with priming (i.e., structural overlap with and without lexical overlap), we also analyzed the relationships between processing of the RRC target sentences (at the regions mentioned above) and the non-RRC prime structures from Tooley (2020). While these analyses cannot tell us about structural priming directly, they do allow us to disclose nonpriming-based explanations of prime–target relationships (such as any adjacent sentences being related to one another in a predictable way). These include the Locative primes, which have a different structure but the same verb as the targets (e.g., *The child pushed by the man to get to the front of the line*), and the Main Clause primes, which have a different structure and verb than the RRC targets (e.g., *The child noticed the man who was going to the front of the line*).

The Locative condition offers a particularly intriguing comparison to the RRC prime conditions because while the locatives have a different structure than the RRC targets, they are identical to the RRC-Same verb primes until the spillover region of the target. This makes them an ideal control condition for ensuring the patterns observed in the other conditions are not due to other (nonstructural) relationships. For the Locatives, we used the verb region (*pushed* in the above example), the by-phrase (*by the man*) and the post-by-phrase “spillover” region (*to get*), which are directly analogous to the regions included for the RRC prime conditions. As this structure is disambiguated from the RRC structure at the spillover region, we predict that any relationships with prime processing and target processing at this critical spillover region will either be nonsignificant or greatly reduced relative to those for the RRC primes (with and without verb overlap).

For completeness and transparency, we also include Main Clause primes. However, the structural regions differ between the Main Clause primes and the RRC targets, and because this condition was considered the baseline condition in the original analyses (reported in Tooley, 2020), no priming effects were expected or observed. We have chosen the most analogous processing regions to use in our control analyses. For the Main Clauses, we used the verb region (*noticed* in the above example), the post-verb region which included the object of the verb (*the man*), which is where the RRC is disambiguated from the main clause structure, and the two-word spillover region after the post-verb region. We included the target verb and by-phrase regions, but not the target spillover region as it is past the point where the structures differed. We predict any processing relationships between primes and targets at these regions will be nonsignificant or greatly reduced from those observed for the RRC primes.

#### Locative control models:

7. Locative-Same Verb **Target Verb Time** ~ Intercept + β_1_PrimeVerbTime + β_2_PrimeBy-phraseTime + β_3_PrimeSpilloverTime + error
8. Locative-Same Verb **Target By-phrase Time** ~ Intercept + β_1_PrimeVerbTime + β_2_PrimeBy-phraseTime + β_3_PrimeSpilloverTime + error
9. Locative-Same Verb **Target Spillover Time** ~ Intercept + β_1_PrimeVerbTime + β_2_PrimeBy-phraseTime + β_3_PrimeSpilloverTime + error

#### Main Clause control models:

10. Main Clause-Different Verb **Target Verb Time** ~ Intercept + β_1_PrimeVerbTime + β_2_PrimePost-VerbTime + β_3_PrimeSpilloverTime + error
11. Main Clause -Different Verb **Target By-phrase Time** ~ Intercept + β_1_PrimeVerbTime + β_2_PrimePost-VerbTime + β_3_PrimeSpilloverTime + error

## Results

### Abstract priming effects: RRC-different verb items

Model 1 revealed that mean-centered prime reading times at the verb (*p* = 0.20), by-phrase (*p* = 0.38), and the spillover (*p* = 0.48) regions did not predict target reading times at the verb region (see Table 1), when primes and targets shared the same structure.

**Table 1:** Model estimates from RRC primes and targets that had different initial verbs and which yielded abstract priming effects in Tooley (2020)

1. Target verb region	Estimate	Standard error	*t* value	*p* value
Intercept	− 1.28	19.49	− 0.065	0.95
Prime verb region	0.050	0.039	1.29	0.20
Prime by-phrase region	0.029	0.033	0.87	0.38
Prime spillover region	0.032	0.045	0.713	0.48
2. Target by-phrase region	Estimate	Standard error	*t* value	*p* value
Intercept	1.76	34.43	0.051	0.96
Prime verb region	0.060	0.053	1.12	0.27
Prime by-phrase region	**0.200**	**0.046**	**4.36**	< 0.001*
Prime spillover region	0.045	0.062	0.73	0.47
3. Target spillover region	Estimate	Standard error	*t* value	*p* value
Intercept	0.630	20.04	0.031	0.98
Prime verb region	− 0.025	0.041	− 0.601	0.55
Prime by-phrase region	**0.120**	**0.035**	**3.30**	0.001*
Prime spillover region	**0.095**	**0.048**	**1.99**	0.047*

**Fig. 1:**
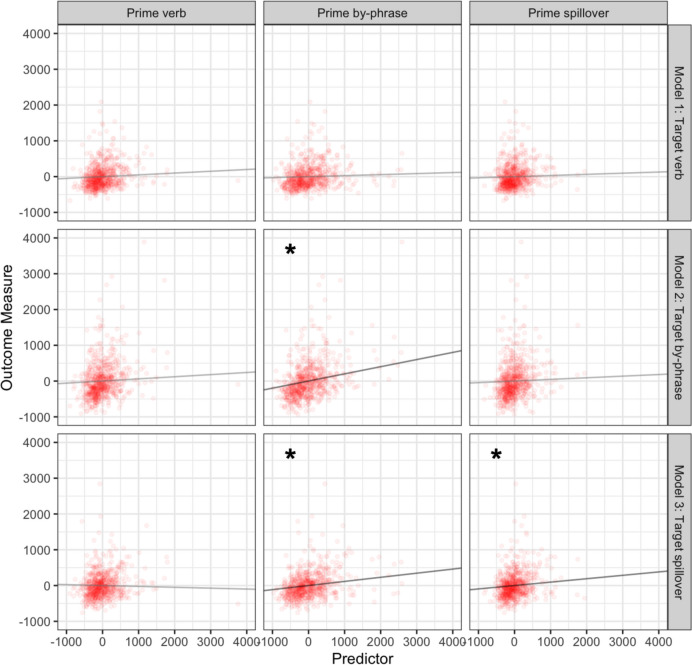
Prime region processing predicted by target region processing for regions associated with abstract priming effects. Each row contains predictors entered into one statistical model. Points reflect observations; lines reflect the estimated unique effect of each variable. Significant relationships are marked with an asterisk

However, Model 2 revealed reading time on the by-phrase region of the primes significantly predicted reading time on the by-phrase region of the targets (*p* < 0.001). Similarly, Model 3 revealed prime reading times at the by-phrase (*p* = 0.001) and spillover (*p* = 0.047) regions significantly predicted target reading times at the spillover region. All effects were positive, such that shorter reading times on prime regions were associated with shorter reading times on target regions. Thus, prime processing at the structurally-difficult portions of an RRC was related to processing of those same target regions, consistent with the idea that experience with a given structure affects subsequent processing of that structure (see Fig. 1).

### Lexical boost effects: RRC-same verb items

**Fig. 2:**
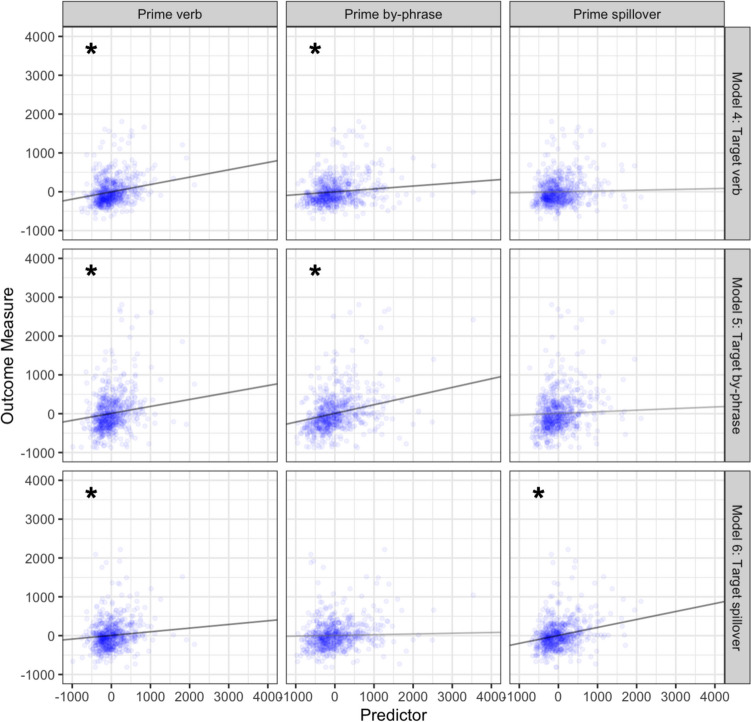
Prime region processing predicted by target region processing for regions associated with lexically boosted priming effects. Each row contains predictors entered into one statistical model. Points reflect observations; lines reflect the estimated unique effect of each variable. Significant relationships are marked with an asterisk

Model 4 showed that prime reading times at verb (*p* < 0.001) and by-phrase (*p* = 0.018) regions (but not the spillover region: *p* = 0.59) significantly predicted target verb reading times. Model 5 showed that prime reading times at the verb (*p* = 0.003) and by-phrase (*p* < 0.001) regions significantly predicted target reading times at the by-phrase sentence region. Similarly, Model 6 revealed that prime reading times on the verb (*p* = 0.041) and the spillover (*p* < 0.001) regions significantly predicted reading times at the target spillover region (see Table 2) (see Fig. 2).

**Table 2:** Model estimates from RRC primes and targets that had *the same* initial verbs and that yielded lexical boost effects in Tooley (2020)

4. Target verb region	Estimate	Standard error	*t* value	*p* value
Intercept	− 1.74	15.32	− 0.11	0.91
Prime verb region	**0.190**	**0.043**	**4.40**	< 0.001*
Prime by-phrase region	**0.075**	**0.031**	**2.38**	0.018*
Prime spillover region	0.021	0.039	0.53	0.59
5. Target by-phrase region	Estimate	Standard error	*t* value	*p* value
Intercept	7.90	23.40	0.34	0.74
Prime verb region	**0.180**	**0.061**	**2.95**	0.003*
Prime by-phrase region	**0.220**	**0.044**	5.03*	< 0.001*
Prime spillover region	0.042	0.055	0.76	0.45
6. Target spillover region	Estimate	Standard error	*t* value	*p* value
Intercept	6.37	16.40	0.39	0.70
Prime verb region	**0.094**	**0.046**	**2.05**	0.041*
Prime by-phrase region	0.019	0.034	0.57	0.57
Prime spillover region	**0.205**	**0.042**	**4.94**	< 0.001*

As before, all effects were positive: shorter prime reading times predicted shorter target reading times (see Table 2), and the structurally critical region of the prime predicted its complementary structural target region. Here, however, the prime verb reading times predicted reading times at all target regions. This implies a boosted structure-to-verb mapping when the same verb appears in both RRC prime and target.

### Locative condition effects

**Fig. 3:**
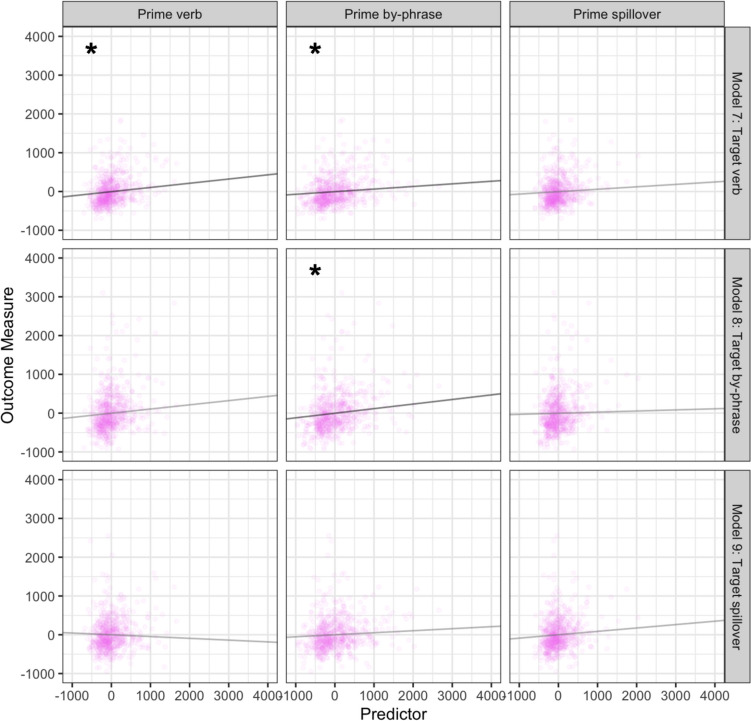
Prime region processing predicted by target region processing for regions associated with locative priming effects. Each row contains predictors entered into one statistical model. Points reflect observations; lines reflect the estimated unique effect of each variable. Significant relationships are marked with an asterisk

Model 7 revealed that prime verb (*p* = 0.016) and by-phrase (*p* = 0.039) reading times significantly predicted target verb reading times, and Model 8 showed prime by-phrase reading times significantly predicted target by-phrase reading time (*p* = 0.012). However, at the spillover region, where the locative and RRC structures are disambiguated, target processing was not significantly predicted by prime reading times (all *p* values > 0.05; see Table 3) (see Fig. 3).

**Table 3:** Model estimates from Locative primes and RRC targets that had *the same* initial verbs

7. Target verb region	Estimate	Standard error	*t* value	*p* value
Intercept	− 4.52	22.23	− 0.20	0.84
Prime verb region	**0.11**	**0.045**	**2.41**	0.016*
Prime by-phrase region	**0.067**	**0.033**	**2.07**	0.039*
Prime spillover region	0.063	0.050	1.26	0.21
8. Target by-phrase region	**Estimate**	**Standard error**	***t* value**	***p* value**
Intercept	− 2.48	33.50	− 0.074	0.94
Prime verb region	0.11	0.065	1.67	0.096
Prime by-phrase region	**0.12**	**0.047**	**2.53**	0.012*
Prime spillover region	0.029	0.072	0.40	0.69
9. Target spillover region	**Estimate**	**Standard error**	***t* value**	***p* value**
Intercept	0.24	28.16	0.009	0.99
Prime verb region	− 0.046	0.054	− 0.84	0.40
Prime by-phrase region	0.052	0.039	1.34	0.18
Prime spillover region	0.087	0.060	1.46	0.15

### Main clause condition effects

**Fig. 4:**
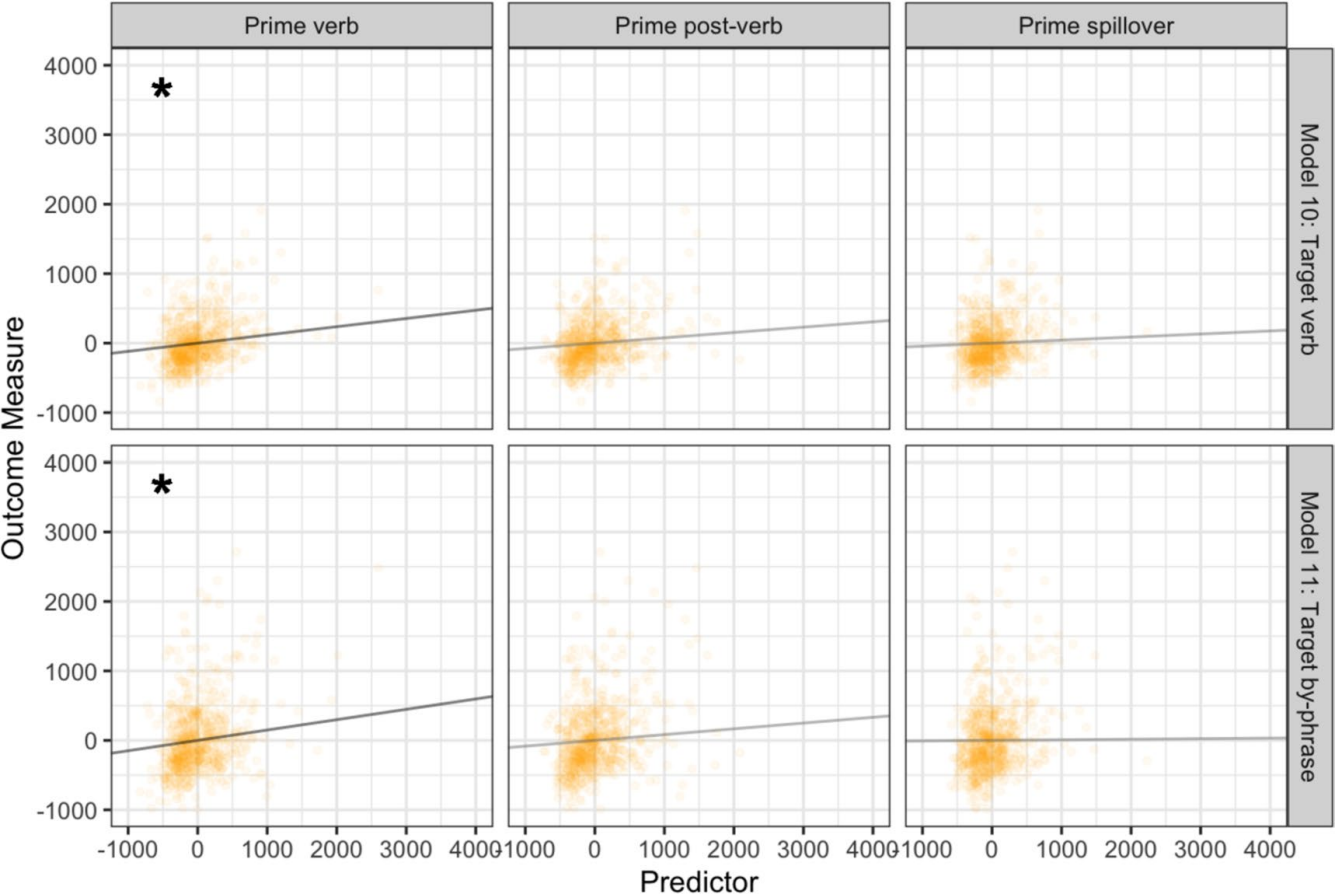
Prime region processing predicted by target region processing for regions associated with main clause priming effects. Each row contains predictors entered into one statistical model. Points reflect observations; lines reflect the estimated unique effect of each variable. Significant relationships are marked with an asterisk

Model 10 revealed prime reading times on the verb region significantly predicted target reading times at the verb (*p* = 0.004), despite different verbs. Model 11 revealed that prime verb reading times also significantly predicted target reading times at the by-phrase (*p* = 0.015). No other significant effects were observed (see Table 4) (see Fig. 4).

**Table 4:** Model estimates from main clause primes and RRC targets that had *different* initial verbs

10. Target verb region	Estimate	Standard error	*t* value	*p* value
Intercept	~ 0	0.022	~ 0	~ 1
Prime verb region	**0.12**	**0.041**	**2.87**	0.0042*
Prime post-verb region	0.077	0.039	1.96	0.051
Prime spillover region	0.063	0.050	1.26	0.21
11. Target by-phrase region	**Estimate**	**Standard error**	***t* value**	***p* value**
Intercept	~ 0	0.36	~ 0	~ 1
Prime verb region	**0.15**	**0.061**	**2.43**	0.015*
Prime post-verb region	0.083	0.058	1.43	0.15
Prime spillover region	0.0081	0.065	0.13	0.90

### Combined analyses

To test whether prime reading times predict target reading times beyond basic relationships of adjacent sentences, we also ran a set of combined models predicting target reading times with significant prime regions from the single-condition models and interactions with sentence condition as predictors. The RRC-Same verb condition was used as a reference level. We report the most relevant effects based on the predicted pattern of larger slopes/effect sizes for lexical *and* structural overlap relative to just lexical or just structural overlap (see Appendix for full model outputs).

At the target verb region, predictors were the prime verb and by-phrase regions and their interactions with condition. Prime verb reading times significantly predicted target verb reading times (*b* = 0.11, *SE* = 0.041, *p* = 0.008), and the slope of this effect was decreased for the RRC-Different verb condition relative to the RRC-Same verb condition, though the interaction did not reach significance (*b* =  − 0.074, *SE* = 0.054, *p* = 0.17).

At the target by-phrase, prime verb and by-phrase reading times and their interactions with condition were included as predictors. Prime verb reading times significantly predicted (*b* = 0.12, *SE* = 0.057, *p* = 0.03), and prime by-phrase reading times marginally predicted (*b* = 0.066, *SE* = 0.039, *p* = 0.097) target by-phrase reading times. There was also a marginal interaction by condition with the RRC-Different verb reading times being less predictive of the target by-phrase than the RRC-Same verb (*b* =  − 0.13, *SE* = 0.076, *p* = 0.098).

At the target spillover region, prime by-phrase and spillover region reading times and their interactions with condition were included as predictors. Prime spillover reading times significantly predicted target spillover reading times (*b* = 0.16, *SE* = 0.041, *p* < 0.001) and marginally interacted with condition such that the slopes of the RRC-Different verb primes (*b* =  − 0.12, *SE* = 0.062, *p* = 0.065) and the Locative primes (*b* =  − 0.085, *SE* = 0.066, *p* = 0.19) were decreased relative to the RRC-Same verb condition, though this trend was not significant for the Locatives. Prime by-phrase reading times marginally interacted with Condition such that the slope for the RRC-Different verb condition was marginally increased relative to the RRC-Same verb condition (*b* = 0.082, *SE* = 0.043, *p* = 0.059).

The overall patterns in the combined-condition models are largely consistent with the single-condition models, though they picked up on fewer “significant” nuanced differences, which could be a power issue. Critically, we again observed that the prime reading times at a sentence region positively predicted processing at that region of the target, and differences in the processing relationships for RRC structures with the same versus different verbs.

## Discussion

The current study used prime sentence reading times to predict target reading times in configurations eliciting abstract priming and a lexical boost in Tooley (2020). Prime reading times predicted target reading times in several sentence regions. When only structure overlapped, processing of structurally critical prime regions predicted processing times for those same regions of the target. When both structure and verb overlapped, prime verb processing predicted processing at all structurally critical regions of the target (see Table 5). Notably, processing on the locative primes, which have a by-phrase and same verb as the targets, did not significantly predict target processing where the structures diverged. Though some patterns were weaker or marginal in the combined analyses, they are consistent with the general idea of structural priming: Structural processing is related to, and likely shaped by, previous structural experience, including experience with particular verbs.

**Table 5:** Significant prime-to-target processing relationships (indicated as *) for prime–target sentence pairs where abstract structural priming effects were observed (top half) and where lexically boosted structural priming effects were observed (bottom half)

Prime to target relationships during abstract structural priming			
Prime region	Target verb region	Target by-phrase region	**Target spillover region**
Verb			
By-phrase		*	*
Spillover			*
Prime to target relationships during lexically boosted structural priming			
Prime region	Target verb region	Target by-phrase region	**Target spillover region**
Verb	*	*	*
By-phrase	*	*	
Spillover			*

Linking prime and target processing offers a new way of investigating the mechanisms of structural priming. We consistently observed significant *positive* prime–target relationships, showing easier prime processing was associated with easier target processing. This is inconsistent with error-driven learning, where greater error signals result in greater weighting changes in favor of the target structure (e.g., Chang et al., 2006; Jaeger & Snider, 2013). The observed positive relationships are consistent with a residual activation account (Pickering & Branigan, 1998) or an associative learning account (e.g., Reitter et al., 2011). In a reading task, this means that successfully parsing the prime RRC structure led to strong activation of the RRC node/representation, which then facilitated parsing of the RRC target. RRC primes where participants initially inferred/activated a main clause interpretation, had longer total reading times and less relative activation of the RRC node/representation, which resulted in less facilitation (and longer reading times) for the RRC targets.

Given the residual activation account has trouble explaining cumulative, long-lived priming (e.g., Fine & Jaeger, 2013; Kaschak et al., 2011), an associative learning mechanism that does *not* rely on an error signal is best able to accommodate all findings. Associative learning models propose use facilitates future reuse when coordinated neuronal firing leads to long-term changes in neuronal connectivity, such as in long-term potentiation and Hebbian learning (see McClelland, 2006, for a review). Associative learning can be modeled computationally through statistical (i.e., Bayesian) or activation-based algorithms (as in ACT-R models). These models have replicated human data patterns of abstract priming and the lexical boost in production (Reitter et al., 2011) and children’s acquisition of syntax (Kidd, 2012). Reitter et al.’s (2011) model also replicated the inverse-frequency effect, whereby lower frequency structures prime more robustly, which is often cited as evidence for error-driven learning (Jaeger & Snider, 2013). Assuming prediction in comprehension relies on covert production (see Pickering et al., 2013), associative learning is a good candidate mechanism for the full range of results.

More intensive examinations of an error-driven learning mechanism are possible with our approach, however. Our data and analyses were limited to total reading times, as this measure provided reliable structural priming effects in Tooley (2020). It is possible that earlier measures could reveal inverse relationships between prime and target processing. Future studies could investigate how processing relationships compare in earlier processing measures if they disclose structural priming. It would also be informative to investigate prime–target processing relationships across primes designed to evoke scaled levels of an error signal, such as nonambiguous full relative clauses versus reduced-relative clauses, which produce a garden-path effect, and so should yield a larger error signal.

In the current study, distinct patterns also emerged for abstract structural priming and the lexical boost of RRCs (see Table 5). When the verb in prime and target differed, prime processing at the verb did not predict target processing. When the prime and target shared a verb, processing of the prime verb predicted processing at all target regions, and prime processing of the critical by-phrase region predicted target processing at the verb. However, these patterns were not consistently reliable in the combined analyses. Cautiously, these differences imply the lexical boost is most likely not a more robust abstract priming effect. Instead, we suggest it is an additional effect alongside abstract priming, rather than the single-mechanism that the residual activation account (Pickering & Branigan, 1998) suggests. A dual mechanism account (e.g., Hartsuiker et al., 2008; Reitter et al., 2011; Tooley & Traxler, 2010) is therefore more parsimonious with our results. The second mechanism could be residual activation (Pickering & Branigan, 1998), explicit memory (e.g., Chang et al., 2006), or some other mechanism. One way to probe this other mechanism would be to investigate prime–target processing relationships across different numbers of intervening sentences to see if patterns change based on the distance between the prime and target, which would be predicted by changes in residual activation (e.g., Pickering & Branigan, 1998).

Most of our analyses tell a straightforward story about structural priming and prime–target processing relationships. The Main Clause results though, where prime verb reading times predicted target RRC verb and by-phrase reading times despite different verbs and nonequivalent post-verbal regions, are surprising. These primes were originally the baseline condition (in Tooley, 2020) and so cannot reflect structural priming. Notably, the verbs in this condition (e.g., *appreciated*, *disliked*) were qualitatively different from those in the lexical overlap conditions (e.g., *pushed*, *squeezed*), which appeared with a by-phrase more often since they occurred in RRC and locative constructions. It is possible, then, that context-specific learning happened whereby having just read an emotion-based verb (such as disliked) in a Main Clause prime (without a by-phrase) and then encountering a physical verb (such as pushed) in the target, set up a strong expectation for a by-phrase in that sentence. This explanation is post hoc but plausible given context-dependent learning effects (Wang et al., 2021). We feel this condition is different enough that its anomalous results can be considered separately from the other conditions.

In conclusion, using processing measures to establish relationships between prime and target sentences offers a novel approach to understanding the nature of structural priming effects and the mechanisms that produce them. Our initial attempt using this approach has revealed positive processing relationships between primes and targets, and that these relationships differ when the verb and structure is shared relative to when just the structure is shared. These observations are most consistent with associative learning accounts that suggest abstract priming and the lexical boost are produced by separate mechanisms. Applying this novel approach could lead to future advancements in our understanding of structural priming.

## Supplementary Information

Below is the link to the electronic supplementary material.

Supplementary file1 (DOCX 15 kb)

## Data Availability

This dataset is publicly available online (https://doi.org/10.18738/T8/A5L2EF).

## Code availability

The R code used to run these analyses and create these figures will be made available upon request from the authors.
