# Supplementary material for: Putting the prime in priming: Using prime processing behavior to predict target structural processing
Source: Psychon Bull Rev. 2025 Jan 30;32(4):1599–610. doi: 10.3758/s13423-025-02643-3 (PMC12325543; doi:10.3758/s13423-025-02643-3)
Supplement: Supplementary file 1 — Supplementary file1 (DOCX 15 kb) [file 13423_2025_2643_MOESM1_ESM.docx]

Appendix: Combined Models Details and Output

RRC Target Verb Region: At the target verb region, the prime verb and by-phrase regions and their interactions with condition (the RRC Same verb condition was reference condition, compared to RRC Different verb and Locative conditions) were predictors.

RRC Target By-phrase Region: At the target by-phrase, prime verb and by-phrase reading times, and their interactions with condition were included as predictors. Both control conditions were included in the model as this is where the MC structure diverges from the RRC structure.

RRC Target Spillover Region: Reading times at the target spillover region were predicted by the prime by-phrase and spillover region reading times and the interaction with these regions and condition (including the RRC Same verb as referent condition compared to the RRC Different verb and Locative conditions).

Table 6: Model estimates for the combined-condition models

| **Target Verb Region** | **Estimate** | **Standard Error** | **t-value** | **p-value** |
| --- | --- | --- | --- | --- |
| Intercept | -3.53 | 18.15 | -.19 | .85 |
| Prime Verb Region | 0.11 | 0.041 | 2.64 | 0.0084* |
| Prime By-phrase Region | 0.035 | 0.027 | 1.28 | 0.20 |
| Prime Verb x Locative | 0.015 | 0.058 | 0.26 | 0.80 |
| Prime Verb x RRC Different Verb | -0.074 | 0.054 | -1.36 | 0.17 |
| Prime By-phrase x Locative | 0.027 | 0.039 | 0.68 | 0.50 |
| Prime By-phrase x RRC Different Verb | -0.019 | 0.039 | -.50 | 0.62 |
|  | | | | |
| **Target By-phrase Region** | **Estimate** | **Standard Error** | **t-value** | **p-value** |
| Intercept | 1.51 | 31.54 | 0.048 | 0.96 |
| Prime Verb Region | 0.13 | 0.057 | 2.20 | 0.023* |
| Prime By-phrase Region | 0.066 | 0.039 | 1.66 | 0.097 |
| Prime Verb x Locative | -0.12 | 0.081 | -1.55 | 0.12 |
| Prime Verb x Main Clause | -0.017 | 0.079 | -0.24 | 0.81 |
| Prime Verb x RRC Different Verb | -0.13 | 0.076 | -1.66 | 0.098 |
| Prime By-phrase x Locative | 0.013 | 0.055 | 0.24 | 0.81 |
| Prime By-phrase x Main Clause | 0.017 | 0.064 | 0.26 | 0.80 |
| Prime By-phrase x RRC Different Verb | 0.054 | 0.055 | 0.99 | 0.33 |
|  | | | | |
| **Target Spillover Region** | **Estimate** | **Standard Error** | **t-value** | **p-value** |
| Intercept | 1.89 | 20.22 | 0.093 | 0.93 |
| Prime By-phrase Region | -0.012 | 0.032 | -0.38 | 0.71 |
| Prime Spillover Region | 0.16 | 0.041 | 3.95 | < 0.001* |
| Prime By-phrase x Locative | 0.045 | 0.044 | 1.02 | 0.31 |
| Prime By-phrase x RRC Different Verb | 0.082 | 0.043 | 1.89 | 0.059 |
| Prime Spillover x Locative | -0.085 | 0.066 | -1.30 | 0.20 |
| Prime Spillover x RRC Different Verb | -0.12 | 0.062 | -1.85 | 0.065 |
